# The Emerging Role of CAR T Cell Therapy in Relapsed/Refractory Hodgkin Lymphoma

**DOI:** 10.3390/jpm12020197

**Published:** 2022-02-01

**Authors:** Jeremy A. Meier, Barbara Savoldo, Natalie S. Grover

**Affiliations:** 1 Lineberger Comprehensive Cancer Center, The University of North Carolina at Chapel Hill, Chapel Hill, NC 27599, USA; Jeremy.Meier@unchealth.unc.edu (J.A.M.); bsavoldo@med.unc.edu (B.S.); 2 Department of Medicine, Division of Hematology, The University of North Carolina at Chapel Hill, Chapel Hill, NC 27599, USA; 3 Department of Pediatrics, The University of North Carolina at Chapel Hill, Chapel Hill, NC 27599, USA

**Keywords:** relapsed/refractory Hodgkin lymphoma, CAR T cells, CD30, immunotherapy

## Abstract

Treatment for Hodgkin lymphoma (HL) has evolved considerably from the time it was originally described in the 19th century with many patients now being cured with frontline therapy. Despite these advances, upwards of 10% of patients experience progressive disease after initial therapy with an even higher percentage relapsing. Until recently there had been limited therapeutic options for relapsed and/or refractory HL outside of highly intensive chemotherapy with stem cell rescue. Improved understanding of the pathophysiology of HL, coupled with the emergence of more targeted therapeutics, has reshaped how we view the treatment of relapsed/refractory HL and its prognosis. With this, there has been an increased focus on immunotherapies that can reprogram the immune system to better overcome the immunosuppressive milieu found in HL for improved cancer cell killing. In particular, chimeric antigen receptor (CAR) T cells are emerging as a valuable therapeutic tool in this area. Building on the success of antibody-drug conjugates directed against CD30, CAR T cells engineered to recognize the same antigen are now reaching patients. Though still in its infancy, CAR T therapy for relapsed/refractory HL has shown exceptional promise in early-stage clinical trials with the potential for durable responses even in patients who had progressed through multiple lines of prior therapy. Here we will review currently available data on the use of CAR T cells in HL, strategies to optimize their effectiveness, and how this therapy may fit into the treatment paradigm of HL going forward.

## 1. Introduction

Hodgkin lymphoma (HL) is a B cell malignancy that affects ~8000 people yearly of all ages with the highest incidence in young adults. Phenotypically, it is characterized by the co-expression of CD15 and CD30 on malignant Hodgkin and Reed–Sternberg (HRS) cells, though it can also be identified by a particular gene signature [1]. HL was one of the first malignancies to show responsiveness to radiation therapy, but treatment for HL has now evolved to include multiagent chemotherapy with 5-year survival rates for all those diagnosed approaching 90% [2]. Despite the success of frontline therapy and the curative potential in HL, upwards of 20–30% will experience disease progression or relapse at some point in their lifetime [3]. Salvage options for treatment in these cases have generally focused on high-dose chemotherapy followed by autologous stem cell transplantation (ASCT), which remains the standard of care to date. However, the emergence of more targeted therapeutics, including anti-CD30 antibody-drug conjugates and immunotherapy, has reshaped how we approach treatment for relapsed/refractory disease [4,5]. Even with these advances there remain a significant fraction of patients who progress, leading to more than 1000 deaths yearly from HL.

A key feature of HL, particularly classical HL (cHL) that we will focus on in this review, is a relatively sparse number of malignant cells interspersed in a heavily immune infiltrated background [6]. In cHL, the immunosuppressive tumor microenvironment (TME) serves a key function in driving cancer cell immune evasion. In patients with progressive disease, strategies for treatment have increasingly focused on immune-based therapies to better target and clear the malignant cells [7]. Chimeric antigen receptor (CAR) T cells have emerged as a novel form of immunotherapy, whereby the patient’s own immune cells are engineered ex vivo to recognize target cancer antigens. CAR T cells have shown exceptional promise in trials for non-Hodgkin lymphoma with even heavily pretreated patients showing high response rates with the potential for durable responses [8]. Given their success in other lymphomas and hematologic malignancies, studies are now evaluating how to improve their efficacy in relapsed/refractory (r/r) HL [9]. Here we will review the currently available data in this area highlighting trials to date, efforts to optimize CAR T efficacy in HL, and how this therapy might fit into the current paradigm of treatment in refractory disease.

## 2. Immune Based Approaches for Treatment of Relapsed/Refractory HL

For years, the mainstay of treatment for r/r HL has been high dose chemotherapy followed by ASCT after this was shown to be a viable therapeutic strategy in the early 1990s [10], with trials demonstrating improved disease-free survival with transplant as compared to chemotherapy alone [11]. While still considered the standard of care if patients are transplant eligible, the success of newer, novel agents has called into question whether transplant is needed in all these patients. Historically, upwards of 50% of patients, particularly those with high-risk disease, will still relapse after ASCT [12]. Attempts at improving relapse and progression-free survival (PFS) in these cases through maintenance therapy after transplant have shown some promise. For example, the AETHERA trial showed that the addition of brentuximab–vedotin (BV, an anti-CD30 antibody-drug conjugate) post-transplant increases the 5-year PFS from 41% to 59% [13]. However, as this was conducted in BV naïve patients, the applicability of these findings in the future is likely limited given the increasing number of HL patients who are seeing BV prior to ASCT, and in some cases, even in the frontline setting [14]. Further assessment of the role of transplant in the management of r/r HL is discussed later in the review. Despite these advances, there still remains a significant fraction of patients who continue to progress through additional lines of therapy or relapse after transplant for whom treatment options remain limited. As treatment of r/r HL in these cases has been extensively reviewed previously [4,15], we will instead highlight here emerging approaches that focus on immune-based therapies for treating HL, particularly CAR T cells.

As mentioned above, a hallmark of cHL is the presence of malignant HRS cells scattered about a large contingent of immune cells which distort the typical lymph node architecture. This supports tumor growth in HL through varied mechanisms. The inflammatory TME fuels the generation of pro-tumorigenic immune and stromal support cells, which modulate the angiogenic, metabolic, and signaling pathways in the tumor environment to promote cancer cell proliferation and survival. The unique chemokine and cytokine milieu here also favors both regulatory T cell formation and recruitment [16], leading to an immunosuppressive niche. This inhibits the activity of cytotoxic T and NK cells, creating further challenges in the effective treatment of r/r HL. Further, single-cell sequencing of the immune repertoire in HL has demonstrated an increase in LAG3+ T-cells [17], with LAG3 being known to mediate tumor tolerance and inhibitory signals downstream of MHC class II binding [18,19]. Similar to this, a high percentage of TIM3+ cells are present in the TME in cHL, with TIM3 known to be an inhibitory receptor and exhaustion marker on T cells [20]. The immunosuppressive TME is also felt to drive therapeutic resistance, whereby the PD-1/PD-L1 axis plays a key role. Looking at the genetic landscape of HL, 97% of patients evaluated in one study had alterations (copy number gain or amplification) in the genes encoding PD-1 ligands [21]. CTLA-4, another immune checkpoint marker, is highly expressed on T cells in the TME in HL as well, while HRS cells and tumor-associated macrophages (TAMs) have elevated levels of the CTLA-4 ligand CD86 that mediates immune suppression [22]. This occurs in conjunction with mutations in HRS cells that affect MHC class I expression, as well as downregulation of CD58, effectively minimizing direct cytotoxicity from CD8 T cells and NK cells, respectively [6]. With this in mind, landmark trials investigating the PD-1 inhibitors nivolumab [23] and pembrolizumab [24] emerged with encouraging results (ORRs 60–70% and CRs 20–30%) in heavily pretreated HL patients and after ASCT failure. CTLA-4 inhibitors, such as ipilimumab, have also shown promise and are being investigated in early clinical trials for refractory disease [25]. Because of the success of these approaches, trials combining immune checkpoint inhibition with chemotherapy or BV have become increasingly common with significant improvements in ORR and CR rates over single-agent therapy alone [26,27]. Given the efficacy of these treatments in the relapsed/refractory setting, immunotherapy-based regimens are now even being applied in the frontline setting [28], with an ongoing cooperative group trial comparing standard frontline treatment with BV and chemotherapy to nivolumab and chemotherapy (NCT03907488).

While the incorporation of immune checkpoint inhibitors has certainly led to improved outcomes, it only addresses one mechanism by which cancer cells escape immune surveillance in cHL. In other hematologic malignancies, especially non-Hodgkin lymphomas (NHL), adoptive T cell therapy has shown the potential for not only high response rates but also durable responses in multiply relapsed and refractory patients. In particular, multiple trials with CAR T cells have repeatedly demonstrated the applicability of this platform for treatment [29,30,31]. CAR T cells make use of the patient’s own immune system and reprogram it through ex vivo genetic engineering to recognize specific cancer antigens. These chimeric molecules are fused with intracellular co-stimulatory and signaling domains that, upon binding of their cognate antigen, drive T cell activation and upregulate their killing machinery. A key advantage of these engineered T cells is that their tumor recognition is independent of HLA, which is a major mechanism of immune evasion of HRS cells. Though the use of CAR T cells for the treatment of HL had lagged behind initially, recent trials show that this is an effective approach for the treatment of relapsed or refractory disease.

## 3. Current State of CAR T Therapy in HL

Early studies demonstrating a potential role for adoptive T cell therapies in the treatment of HL took advantage of the fact that 30–40% of cHL cases are also Epstein–Barr Virus (EBV) positive, with HRS cells expressing EBV antigens [32]. This led to the development of therapies aimed at expanding ex vivo autologous cytotoxic T cells targeting these antigens [33,34,35,36]. Based on the results of these preclinical experiments and early phase trials that showed response rates upwards of 50%, there was significant interest in generating CAR T cells that could be more widely applicable to all HL patients, regardless of their EBV status.

The success of BV in r/r HL also provided a nidus to use CD30 as the target for CAR in HL. CD30 has the added advantage of being highly expressed on malignant HRS cells and minimally expressed on other cells and tissues, thereby limiting the potential for toxicity. Wang and colleagues were the first to publish on the feasibility and tolerability of anti-CD30 CAR T cells for the treatment of r/r HL [37], where they treated 18 patients on a Phase 1 trial. The CAR construct used in their study was co-expressed with a CD137 (4-1BB) co-stimulatory domain and delivered with a lentiviral vector. Patients included in the study were heavily pre-treated, though few had seen BV or checkpoint inhibitors prior. As part of the study design, they were able to receive any of three potential conditioning regimens from more standard lymphodepletion with fludarabine and cyclophosphamide to a more disease-controlling regimen with gemcitabine, mustargen, and cyclophosphamide. Patients could have also received a regimen of paclitaxel and cyclophosphamide. They demonstrated in this cohort an ORR of 39% (all partial responders) with 28% of patients showing stable disease at two months after CAR T cell infusion with a median PFS of 6 months. Based on the dose-escalation protocol, patients received anywhere from 1.1 × 10^7^ up to 2.1 × 10^7^ cells/kg. Peak levels of CAR T cells were detected 3 to 9 days after infusion with levels returning to nearly undetectable at 8 weeks. There was however evidence of CAR T cells reaching the tumor microenvironment based on IHC staining of tumor specimens suggesting T cells likely persist here longer than those in circulation. The treatment was well tolerated with no episodes of Grade 3 or 4 cytokine release syndrome (CRS) or neurotoxicity. All patients experienced Grade 1 or 2 febrile syndrome (maybe CRS), around the time of infusion that quickly resolved without intervention. Low levels of CRS seen in this study and subsequent trials are in contrast to what has been seen to date with other CAR T cell-based approaches, particularly anti-CD19 based CAR therapies. The reason for this is likely multifactorial, including but not limited to inherent differences in the target antigen and disease burden, especially when considering the relatively low numbers of HRS cells in HL.

The safety of anti-CD30 CAR T cells was further demonstrated in another Phase I trial conducted by Ramos and colleagues [38]. Here, nine patients with relapsed or refractory HL or anaplastic large cell lymphoma underwent treatment with up to 2 × 10^8^ CAR T cells/m^2^. The CAR construct in this study differed from that previously used by the group in China as it was delivered to T cells using a gammaretrovirus and with a CD28 co-stimulatory domain. Additionally, although no lymphodepletion was used ahead of CAR T cell delivery, secondary endpoint analysis showed an ORR of 33% (including durable responses) highlighting the direct effect of CAR T cells. Similar to the results of Wang et al., peak levels of CAR T cells were seen one week after infusion, and by 6 weeks, the CAR signal was minimally detectable in blood. There was also no appreciable toxicity from the CAR T cells, including no CRS.

Based on these results, our group, in conjunction with the Center for Cell and Gene Therapy (Baylor College of Medicine), initiated parallel Phase I/II trials further investigating the efficacy of anti-CD30 CAR T cells in r/r HL [39]. A total of 41 patients were enrolled and the cohort consisted of heavily pre-treated individuals (median seven prior lines of therapy), including many that had seen BV, checkpoint inhibitors, and had undergone an autologous transplant. Most patients underwent a lymphodepletion regimen containing fludarabine (bendamustine–fludarabine or cyclophosphamide–fludarabine), though 8 of the 42 patients received bendamustine alone. The CAR construct was the same as that used by Ramos et al. where T cells were transduced using a gammaretroviral vector with a CD28 co-stimulatory domain. The primary endpoint was safety with no dose-limiting toxicities seen in patients at even the highest dose level for treatment (2 × 10^8^ cells/m^2^) with the most common adverse effect being cytopenias primarily related to lymphodepletion (grade 3/4 thrombocytopenia in 24% of patients and grade 3/4 neutropenia in 10%). CRS was limited to grade 1 and did not require intervention, and similar to prior studies, there was no neurotoxicity observed. ORR of those evaluable for a disease response was 72% with ~60% of these patients demonstrating a complete response. The one-year PFS and overall survival rates were 41% and 94%, respectively. Moreover, more than a third of the patients showed durable responses to the treatment with five patients remaining in a CR more than a year out from infusion. Based on these results, a multi-center phase 2 pivotal trial of CD30 CAR T cells for relapsed/refractory HL is currently ongoing (NCT04268706).

## 4. Avenues for Optimizing CAR T Therapy in HL

**Figure 1:**
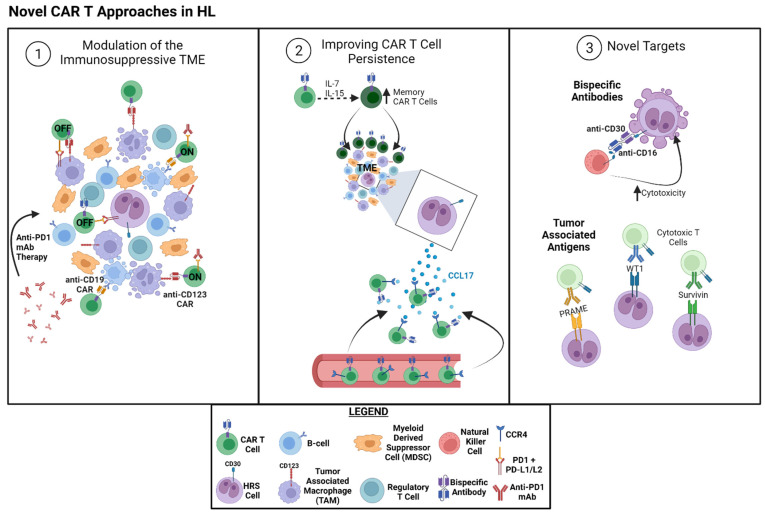
Novel CAR T approaches in HL: Emerging strategies for optimizing CAR T therapy include the following: (1) *Modulation of the Immunosuppressive TME*—the hallmark of classical Hodgkin lymphoma is relatively few malignant Hodgkin Reed–Sternberg (HRS) cells interspersed in an immune infiltrate dominated by immunosuppressive cells, including regulatory T cells, tumor-associated macrophages (TAMs), and myeloid-derived suppressor cells, among others. As these immune cells represent the bulk of the tumor, therapies targeting the TME could improve treatment response in cases of relapsed and/or refractory HL. To date, this has included CAR T products targeting TAMs (anti-CD123), as well as regulatory B-cells (anti-CD19), in the TME. The PD-1/PD-L1/L2 axis represents a critical mechanism that leads to CAR T dysfunction and combined use of checkpoint inhibitors may allow for a more prolonged anti-tumor effect. (2) *Increased CAR T cell Persistence*—Ongoing tumor control requires that CAR T cells be successfully recruited to the TME in order to carry out their function. HRS cells are known to secrete high levels of CCL17, which is generally used to recruit immunosuppressive cells. CAR T cells engineered to express the receptor to CCL17 (CCR4) may allow for increased penetration of CAR T cells into the TME, leading to enhanced antitumor activity. Memory T cells have been shown to persist longer than traditional effector cells and strategies to enhance their formation, including the cytokine milieu (i.e., IL-7 and IL-15) that is used for their ex vivo expansion, may impact the number of infused memory cells. (3) *Novel antigens*—Natural killer (NK) cells are emerging as an alternative to traditional T cells for the introduction of the chimeric antigen receptor. Supporting the efficacy of NK cells for this purpose, studies have demonstrated that a CD16/CD30 bispecific antibody that re-engages NK cells to target HRS cells can lead to tumor regression in relapsed/refractory HL patients. While HRS cells do not appear to experience antigen loss as a mechanism of CAR T cell resistance as of yet, novel targets may allow for the development of other CAR T cells or even multiantigen targeted CAR T cells to improve responses in patients. Similar to this idea, cytotoxic T cells expanded ex vivo to recognize tumor-associated antigens, such as WT1, PRAME, or Survivin, have shown promise in treating HL. Figure created with Biorender.com.

The above results highlight the potential of CAR T treatment in even the most resistant HL patients. Building on these early clinical studies, there are now upwards of 20 clinical trials in progress further investigating the role of CAR T cells in the treatment of relapsed/refractory HL (recently reviewed in [40]). As the use of CAR T therapy in HL is still in its relative infancy, questions remain as to which patients it should be considered for, and how one might further improve on the design and development of CAR T cells to maximize its benefit. In the following sections, we will discuss recent data on strategies to uncover the full potential of this therapy in HL, which is summarized in Figure 1.

### 4.1. Predicting Patient Response to CAR T Therapy

A recent study by our group evaluated factors that were associated with response to anti-CD30 CAR T cells in an attempt to develop a predictive model of responders [41]. Looking at metabolic tumor volume (MTV), which is a measure of tumor burden using ^18^F-FDG PET imaging based on the maximum standardized uptake value, we noted a direct correlation between MTV ahead of CAR T treatment and PFS. Patients with higher MTV (greater than 60 mL) prior to lymphodepletion and cell infusion had a significantly lower 1-year PFS (14%) as compared to those with a low MTV (58%). Patients who responded to bridging therapy with a decrease in MTV from high to low ahead of CAR T cell infusion also had an improved 1-year PFS (40%) compared to those who showed no reduction in tumor burden with bridging therapy (1-year PFS 0%). That being said, bridging therapy in itself was not associated with a statistical difference in PFS. Surprisingly, the degree of expansion and/or persistence of anti-CD30 CAR T cells did not lead to differences in PFS in this cohort. Similarly, there was no difference seen among the MTV groups in pre-CAR T cytokine levels or peak cytokine levels after cell infusion. The percentage of circulating PD-1^+^ T cells was not predictive of response to treatment, though the study was unable to characterize the extent to which PD-1^+^CAR T cells specifically were present.

### 4.2. Targeting the Tumor Microenvironment

The above results indicate that MTV may be a useful parameter to risk stratify potential CAR T candidates in HL and that bridging therapy to lower MTV ahead of lymphodepletion might lead to improved outcomes. Given the limited number of malignant HRS cells in the TME that is characteristic of cHL, the authors speculated that the uptake seen in PET imaging may be more reflective of the immunosuppressive niche supporting cancer growth. Consistent with this, a recent report demonstrated that cells from the myeloid lineage take up the most glucose in the TME [42]. This is particularly intriguing as myeloid-derived suppressor cells (MDSCs) are known to rely on a glycolytic transcriptional program to carry out their function, which impacts immune function in the TME [43]. Similarly, tumor-associated macrophages (TAMs) represent another major myeloid-derived cell component in the TME that has a high glycolytic capacity [44], and the percentage of TAMs in the tumor has been associated with decreased overall survival in HL [45]. As the TME is a key factor in the development and progression of cHL, TAMs could represent another potential target for CAR T development. For example, Ruella and colleagues explored this strategy in a pre-clinical model of HL by targeting CD123, a molecule found on malignant HRS cells and TAMs, with CAR T cells [46]. Anti-CD123 CAR T cells not only eradicated HL in mouse xenograft models but also led to the generation of long-lived memory cells. When mice which had previously received anti-CD123 CAR T cells were re-challenged with HL cells, there was no appreciable tumor growth, while there was significant re-expansion of the CAR T cells pointing to the potential for CAR T cell persistence and long-lasting immune surveillance even when using other CAR targets.

An alternative way to target the TME in HL patients was tested by Svoboda and colleagues, who used CAR T cells directed against CD19 in an attempt to deplete potentially immunosuppressive B cells from the TME in HL [47]. B cells have been implicated in both anti-tumorigenic and pro-tumorigenic roles, with the latter arising from the action of regulatory B cells that drive IL-10 production to limit immune cell activation. B cells have also been reported to contribute to cancer immune evasion by driving chronic inflammation and immune complex formation that leads to increased activity of immunosuppressive M2-like tumor-associated macrophages [48]. The other potential advantage of using CD19 CAR T cells here is that they may also target putative CD19 positive HRS clones that have been shown to circulate [49]. Five patients were evaluated in this study utilizing T cells that instead of being virally transduced to express the CAR, were electroporated to deliver messenger RNA. This strategy allowed for transient expression of the CAR with the rationale that short-term expression would effectively clear target B cells, while also minimizing toxicity. In their cohort of heavily pre-treated patients, they had a 40% response rate, including one complete response. Reported toxicities were attributed to the lymphodepletion regimen rather than the CAR T cells themselves. Additional studies are underway by this group evaluating whether a more traditional, virally transduced CD19 CAR may be effective in the treatment of r/r HL.

The above results highlight what has been studied to date in terms of targeting the TME in HL, though it represents only a subset of the immunosuppressive cells known to be found here. More research is needed to clarify the impact of targeting other pro-tumorigenic cells in the TME, such as cancer-associated fibroblasts, regulatory T cells, or MDSCs, among others. MDSCs are particularly intriguing as the degree to which circulating MDSCs are found in HL correlate negatively with clinical outcome [50], and MDSCs are known to inhibit the activity of CAR T cells [51]. Targeting MDSCs then might prove to be valuable therapeutically, an approach that has been efficacious in other tumor types, such as breast cancer [52].

### 4.3. Improving CAR T Cell Persistence

The issue of persistence of CAR T cells is of paramount importance to their efficacy and in order to achieve durable responses in patients. To date, the clinical trials using anti-CD30 CAR T cells have shown detectable levels of circulating CAR T cells up to 8 weeks post-infusion (in rare patients they are detected months out), though they may exist for longer in the TME and tissues [37,38,39]. In some patients, it is conceivable that this immune surveillance may not be sufficient for the complete eradication of the tumor cells. This has resulted in increased interest in ways to improve the longevity of CAR T cells after infusion.

One approach that has been applied to CAR T development for HL has been the use of an infused product that is enriched for memory stem CAR T cells. Because of the gene manipulation required for CAR expression, most CAR T cells consist of a population predominately made up of terminally differentiated effector cells. While these cells have excellent in vitro anti-tumor properties, they are often not as effective in vivo [53] owing to increased exhaustion and altered T cell homing. More recent studies have shown that less differentiated adoptively transferred T cells, especially a subset of memory T cells termed memory stem T (T_scm_) cells, have the highest antitumor potential [54,55,56]. The advantage offered by this subset may also apply to HL, as suggested by a recent report from Alvarez-Fernandez et al. [57]. They showed that anti-CD30 CAR T cells enriched with T_scm-like_ cells had superior anti-tumor activity in a mouse xenograft model of HL compared to cell products lacking T_scm_ cells. Further, T_scm-like_ cells were resistant to exhaustion in the presence of repeated antigen stimulation, and they were capable of preventing tumor growth in a re-challenge model. Strategies aimed at increasing the population of T_scm_ cells could provide a therapeutic advantage over traditional CAR platforms, though it remains unclear how best to generate these cells.

A potential mechanism for increasing the memory pool of these cells may lie in the design of the CAR T cell. When evaluating the impact of co-stimulatory domains in CAR T cells on their phenotype, it was noted that those that express 4-1BB (CD137) were more likely to be skewed towards central memory cells [58]. This was in contrast to those that expressed CD28 as their co-stimulatory domain, which tended to be more effector-like. Cells expressing 4-1BB were found to persist longer than their CD28 counterparts. This difference was attributed to distinct metabolic pathways favored by each cell type, which was felt to be key in determining their function. Whether this is similarly seen in the case of CD30 CAR T cells remains to be determined. Though data are limited, early phase clinical trials to date using either 4-1BB [37] or CD28 [38,39] for the co-stimulatory domain seem to show a comparable duration of detectable CAR T cells in the peripheral blood. A recent study did demonstrate the value in optimizing the CD30 CAR co-stimulatory domain, as those CD30 CARs containing two co-stimulatory molecules (CD28 and OX40) were longer lived and had greater anti-tumor action [59].

The choice of agent used for lymphodepletion may also play a role in CAR T memory cell development. Studies have shown that fludarabine can lead to increased memory formation in both CD4 and CD8 T cell compartments [60]. In our anti-CD30 CAR T trial for r/r HL patients, we noted that those patients who received fludarabine as part of their conditioning regimen showed higher circulating levels of IL-7 and IL-15 than patients who did not receive fludarabine [39]. These cytokines have been linked to steering naïve T cells to form memory T cells [61], and considering this, it was not surprising that those patients who received fludarabine also experienced longer CAR T cell persistence in the trial. Alternative strategies aimed at increasing IL-7 production could be useful then in increasing the memory pool of these cells with one approach being coupling IL-7 gene expression with the CAR, which in pre-clinical studies has been shown to lead to an increased population of memory CD8 T cells [62].

### 4.4. Enhancing CAR T Trafficking

Another way to improve the efficacy of CAR T cells in HL is to increase their trafficking to the TME. One barrier to CAR T cells gaining access to the malignant HRS cells in HL is the immunosuppressive chemokine and cytokine milieu that they encounter. CCL17/TARC represents one of the key chemokines here, which is important for recruiting Th2 and regulatory T cells that express the cognate receptor CCR4, which further fuels the immunotolerant background. Cytotoxic T cells do not express CCR4 and, therefore, are not readily recruited to the TME in HL. Pre-clinical work demonstrated that forced expression of CCR4 on adoptively transferred T cells improved their activity and tumor-killing potential [63]. This has led to the current clinical trial at the University of North Carolina Lineberger Cancer Center (NCT03602157) evaluating anti-CD30 CAR T cells that co-express CCR4 for treatment of r/r HL or CD30+ cutaneous T cell lymphoma [64]. Preliminary results from the Phase 1 dose-escalation trial included eight HL patients who were evaluable for disease response. All patients in the trial received bendamustine and fludarabine for lymphodepletion. No dose-limiting toxicities have been seen to date. All eight patients responded to treatment with 75% having a complete response with the median PFS not having been reached after a median follow-up of 12.7 months. Moreover, there was significant enrichment of CAR T cells at the tumor site (14.4 × 10^5^ copies/µg of DNA) compared to the peripheral blood (4.3 × 10^5^ copies/µg of DNA). This suggests that with the proper modifications, CAR T cells in HL can be further refined to improve their efficacy and potentially, the durability of their response.

### 4.5. NK Cell-Based Therapeutic Approaches

Among the multiple mechanisms to evade the immune system employed by HRS cells, CD58 downregulation represents a common feature in HL patients who relapse [65]. CD58 is generally recognized by NK cells, as well as cytotoxic T cells, via binding to the CD2 receptor. When downregulated, these effector cells cannot be fully engaged. Bispecific antibodies are one well-studied means to re-engage the immune system to recognize tumors. For HL, the AFM13 trial utilized a bispecific antibody targeting CD16 on NK cells fused to an antibody recognizing CD30 to activate NK cells and bring them into close proximity to HRS cells for direct cancer cell killing [66]. This Phase I trial showed both the safety of the therapy and the clinical activity of the antibody, with 60% of the 28 patients enrolled experiencing disease control with a median time to next therapy of 5 months. From a cellular therapy standpoint, these results prompted researchers to combine the infusion of the AFM13 bispecific antibody with a purified population of allogeneic, cord-blood derived NK cells with the combination product showing enhanced anti-tumor activity in pre-clinical models of HL [67]. Utilizing ex vivo expanded NK cells in conjunction with the AFM13 antibody allowed for the activation of NK cells prior to infusion, while also permitting the modulation of the NK cells to be shifted to a memory phenotype based on the cytokine milieu they were subjected to in culture.

This therapy has since been moved to Phase 1 clinical trials at MD Anderson (NCT04074746) with preliminary results presented at AACR in 2021, showing a 100% response rate in r/r HL in the four patients evaluable at the time of presentation. The other advantage of this therapy is that theoretically, the NK cells can be isolated from healthy donors for an “off-the-shelf” product. In this way, it could be delivered to patients without the same wait time usually required for manufacturing autologous products or the concern about prior therapies impairing immune cell function. However, a potential limitation lies in the relative fragility of NK cells and the challenges that could come with freezing and thawing these products from a viability standpoint. The possibility of using other immune cells outside of T cells as the backbone for cellular therapy opens the door to novel approaches for improving these treatments for patients with HL. In line with this, CAR NK cells have been shown to be effective in the treatment of non-Hodgkin lymphomas (NHL), and potentially with a more favorable side effect profile [68].

### 4.6. Combined Checkpoint Inhibition and CAR T Cell Therapy

Interestingly, there has been no evidence of CD30 antigen loss in HL patients who relapse after CAR T therapy [39]. This is contrary to what is seen in NHL patients where CD19 epitope loss is one of the mechanisms of antigen escape [69]. Nevertheless, relapse does occur in some HL patients suggesting that the effectiveness of anti-CD30 CAR T may still be impaired by other mechanisms. One such mechanism is the retained expression of PD-1, which engages PD-1 ligands that have been upregulated both by HRS cells and by immunosuppressive cells in the TME, particularly TAMs [7,9]. In a study evaluating the role of checkpoint inhibitors in patients who progressed following CAR T treatment, researchers observed a clinical benefit with all five patients responding, including those that had not responded to checkpoint inhibition ahead of CAR T therapy [70]. The median time from CD30 CAR T cell infusion to checkpoint inhibitor treatment in this study was 132 days, so while less likely, it is intriguing to speculate that some of the responses observed were secondary to reprogramming and reactivating CAR T cells that had persisted after the initial infusion (presumably memory cells). The efficacy of immunotherapy after CAR T failure is continuing to be explored in a Phase 1 trial at our institution (NCT04134325).

One wonders, though, whether an upfront combination of checkpoint inhibition and CAR T cell infusion may prove to be a more effective strategy, as is currently being explored in solid tumors [71]. In fact, a recent trial in HL looked at exactly this approach, though instead of CAR T cells, the authors utilized polyclonal autologous or allogeneic T cells expanded ex vivo to recognize a number of novel target antigens in HL, including WT1, Survivin, and PRAME [72]. They coupled these infused adoptive T cell products with nivolumab treatment both pre- and post-T cell infusion (in most patients), which continued until either disease progression or immune-related events were seen. There was no dose-limiting toxicity seen in the study and of the eight patients who had active disease, 50% had at least stable disease one year out from treatment. Nivolumab treatment was associated with increased persistence of these T cells recognizing tumor-associated antigens, pointing to the potential utility of combining these therapies.

## 5. Interplay of Transplant and CAR T Therapy

While the role of adoptive T cell treatments is becoming clearer in relapsed/refractory HL patients who are transplant-ineligible or relapse after transplant [73], it remains to be determined how best to integrate CAR T cells into those patients where transplant remains an option. As discussed above, autologous stem cell transplant remains the standard of care for r/r HL patients. Patients who have a complete response ahead of transplant with salvage therapy or who remain chemosensitive, tend to have the best long-term outcomes [74,75,76,77]. However, up to 50% of patients relapse after auto transplant. In those that have relapsed, allogeneic transplant currently represents the option with the highest curative potential [78], as more novel approaches (anti-CD30 antibody-drug conjugates, such as BV, checkpoint inhibitors, etc.) are generally less likely to result in long-term disease control. That being said, there are occasional durable remissions seen with these novel agents, and given the significant morbidity associated with allogeneic transplant, these alternative therapies are often trialed first. Notwithstanding the toxicities of transplant, long-term follow-up of patients receiving allogeneic SCT for r/r HL demonstrates the efficacy of the approach with 5-year OS rates of 51.4% and relapse-free survival of 38.9% [79]. Though enthusiasm for allogeneic transplant has been hampered historically by the high morbidity from transplant-related complications [80], reduced-intensity conditioning regimens have led to improved rates of non-relapse-related mortality (NRM) [81]. Meta-analysis has also shown that outside of an improvement in NRM, the advances in allogeneic transplant management over the past 20 years has resulted in an increase in 1-year OS from 54% to 80% [82]. Similar to autologous transplant, those patients with chemosensitive disease continue to show the best outcomes in allogeneic transplant with overall survival at 1 year further improving to 96%. Given the importance of minimal residual disease ahead of transplant in these studies and the success of CAR T therapy in achieving complete responses in even heavily pretreated patients, one could imagine a role for CAR T therapy as a bridge to transplant in select patients.

This has yet to be explored in HL, though is being investigated in cases of B-cell acute lymphoblastic leukemia (B-ALL). In a study of B-ALL patients who had undergone allogeneic transplant, outcomes for those patients who had received CAR T cells as a bridge to transplant compared similarly to those receiving chemotherapy when looking at NRMrates, leukemia-free survival, and overall survival [83]. Patients receiving CAR T cells ahead of transplant did however have a higher incidence of acute graft versus host disease (grade II only) and chronic graft versus host disease, highlighting a potential negative effect of modulating the immune system ahead of transplant. However, a similar study in B-ALL and NHL patients did not show any increased toxicity from allogeneic transplant in patients who had previously received CAR T therapy [84]. Multiple studies evaluating checkpoint inhibitor therapy, which likely has similar immunomodulatory effects if not more than CAR T cells, ahead of transplant have also not shown any deleterious effect. A study by our leukemia group showed that there was no difference in overall survival, relapse-free survival, or incidence of graft versus host disease in acute myeloid leukemia (AML) patients who had been treated with pembrolizumab prior to allogeneic transplant [85]. In some cases, immune reprogramming ahead of transplant may lead to even better outcomes. Herrera and colleagues demonstrated that heavily pre-treated patients who received PD-1 therapy ahead of autologous stem cell transplant and responded had improved rates of progression-free survival at 18 months compared to PD-1 non-responders (86% vs. 51%, [86]). Overall, more research is needed in order to clarify the potential benefits (i.e., increased graft vs. lymphoma effect) and risks (increased GVHD) associated with the use of CAR T cells and other immunomodulatory agents, such as PD-1 inhibitors, ahead of transplant [87]. However, based on the possibility of durable remissions with CAR T cell therapy in HL, as seen in early phase trials, transplant is currently being reserved for future relapses in order to avoid the potential morbidity from transplant-related complications.

Another use of CAR T cells in the context of transplant could be as a maintenance or consolidation strategy post-transplant in r/r HL. This is currently under investigation at our institution (NCT04083495) where high-risk patients (defined by lack of CR after initial treatment, CR < 12 months, or extranodal disease at time of pre-transplant salvage) received an infusion of CD30 CAR T cells after ASCT [88]. A total of 15 patients received CAR T cells on this Phase 1 trial at a median of 22 days post-transplant with the treatment being well tolerated (no grade 2 or higher CRS, no neurotoxicity) with hematologic toxicity the most commonly seen (grade 3/4 cytopenias in 40% of patients). The PFS at 1 year in HL patients was 79% with an OS of 100%. This is at least comparable, if not superior to, the results seen in the AETHERA trial where BV was used as a maintenance strategy post-transplant [13]. As discussed above, that trial only included BV naïve patients, which may not be as applicable in today’s treatment landscape where many patients are seeing BV earlier in their treatment course. In fact, in the NCT04083495 trial, 80% of patients had been treated with BV at some point prior to transplant. Based on the results of this Phase I trial, CAR T cells may serve an important role in the post-transplant setting, particularly in patients previously receiving BV who may have progressed through this therapy or have neuropathy which prevents them from being able to receive further BV.

There have been questions regarding the safety of CAR T therapy after both autologous and allogeneic transplants with concerns regarding an increased incidence of immune-related adverse events. This has, however, not been appreciated to date with studies largely demonstrating the long-term safety of CAR T cells even after transplant [89]. This data is largely in CD19 targeted CAR T cells, though we anticipate that the same will be observed for other CAR T cells, including anti-CD30 CARs. Data from our Phase I/II clinical trial with CD30 CAR T cells [39] supports this as 90% of patients had received either an autologous or allogeneic transplant at some time in their treatment history prior to receiving CAR infusion. There was no apparent increase in toxicity in these patients at a median follow-up of 533 days demonstrating the safety of combining transplant and CAR T therapy.

The success of CAR T therapy in hematologic malignancies has also called into question whether this treatment option could replace stem cell transplant as the standard of care in the future for relapsed or refractory disease [90]. In an intent to treat analysis, Dreger et al. demonstrated in cases of r/r NHL that CAR T cells were not inferior to allogeneic stem cell transplant with comparable rates of PFS (39% vs. 33%) and OS (68% vs. 54%) at 12 months [91]. CAR T cell therapy carried the added benefit of a significant reduction in non-relapse mortality (2% as compared to 21%), though patients receiving CAR T cells did have a higher tendency for relapse (59% vs. 44%). Whether this holds true in HL patients is unclear, and more data are needed to confirm these results before reshaping how we think about the treatment of relapsed/refractory disease.

## 6. Concluding Remarks

Though the majority of patients diagnosed with HL will respond to frontline therapy and be cured, a sizeable fraction (up to 30%) may relapse and a number of patients may have refractory disease. Treatment options in the relapsed and/or refractory setting have changed dramatically since the turn of the century with a number of novel agents revolutionizing our approach to treatment. CAR T cells have now joined the armamentarium for treatment of r/r HL and demonstrate exceptional promise with high response rates in this population, along with the potential for durable responses. How this therapy fits with the current treatment paradigm in r/r HL is the key question going forward. The fact that there has been limited toxicity observed with anti-CD30 CAR T cells to date is especially encouraging and may cause us to rethink how we view the timing and role of transplant for r/r HL in the future. That being said, much work remains to determine how best to optimize this therapy and whether or not the durable responses we have seen thus far truly translate to a ‘cure’ for these patients. Further clarification of the optimal target for CAR T cell design in HL is also needed, especially when considering that the majority of cells encountered are not malignant HRS cells, but rather immunosuppressive cells of the adaptive and innate immune systems. Combinatorial treatment approaches that address both of these populations may be needed to fully eradicate the tumor. The success of this will ultimately dictate the degree to which CAR T cells can be relied upon for treatment in r/r HL. The results from early-phase clinical trials to date would suggest that the future here is bright and that even the most refractory patients could see long-term disease control.
